# Relationship between long working hours and periodontitis among the Korean workers

**DOI:** 10.1038/s41598-017-08034-6

**Published:** 2017-08-11

**Authors:** Wanhyung Lee, Sung-Shil Lim, Byurira Kim, Jong-Uk Won, Jaehoon Roh, Jin-Ha Yoon

**Affiliations:** 1 The Institute for Occupational Health, College of Medicine, Yonsei University, Seoul, Korea; 2 Graduate School of Public Health, College of Medicine, Yonsei University, Seoul, Korea; 3 Incheon Worker’s Health Center, Incheon, Korea; 4 Severance Hospital, Health System, Yonsei University, Seoul, Korea; 5 Severance Dental Hospital, College of Dentistry, Yonsei University, Seoul, Korea; 6 Department of Preventive Medicine, College of Medicine, Yonsei University, Seoul, Korea

## Abstract

We aimed to investigate the association between long working hours and periodontitis, and whether such an association constitutes an exposure-response relationship. Data for this study were collected from the Korea National Health and Nutrition Examination Surveys conducted from 2007 to 2014; 17,533 workers (9,483 of men and 8,050 of women) were included. The odds ratios (OR) and 95% confidence intervals (CI) for the analysis of periodontitis defined as positive of Community Periodontal Index in relation to working hours were calculated using multiple logistic regression models with various stratifications. Compared to participants who worked ≤40 hours per week, the prevalence ratio (95% CI) for the periodontitis was 1.19 (1.14–1.24) and full adjusted OR (95% CI) was 1.09 (1.02–1.18) in participants who worked over 40 hours per week. The OR (95% CI) for periodontitis were 1.09 (0.99–1.19) in working group of 40< and ≥52 and 1.10 (1.01–1.20) in working group of >52 hours per week with a significant trend (p = 0.0233) even after adjusting for age, socioeconomic status, healthy behaviour, chronic disease, and dental care status. Long working hours are associated with periodontitis among Korean workers in an exposure-response manner.

## Introduction

The worldwide economy is increasingly based on market globalization and online commerce. Therefore, the workplace environment has rapidly changed, and more people are employed in non-standard workplaces. Many individuals are made to work for longer hours with complex work schedules to suit the ‘never-sleep society’^1^. As a result, employment has become more precarious, and many occupational health professionals are concerned that undesirable work environments are closely linked to negative health effects.

Long working hours (LWH) are particularly deemed to be hazardous to workers’ health. To prevent excessive workloads, the International Labour Organisation and the European Directive on Working Hours introduced specific standards for work patterns and hours as below; (i) No more than 48 hours a week averaged over a 17 week period, (ii) A minimum daily rest period of 11 consecutive hours, (iii) A minimum weekly rest period of 24 or 48 consecutive hours averaged over 14 days, (iv) A minimum of 20 minutes rest in any work period of more than 6 hours, (v) A maximum of 8 hours night work every 24 hours averaged over a 17 week period, (vi) Free health assessments for night workers, (vii) Paid annual leave of at least 4 weeks^2^. Nevertheless, according to a survey by the Organization for Economic Cooperation and Development (OECD), LWH are still common in many countries including Greece, Mexico, Japan, and South Korea^3^.

LWH are a critical risk factor for fatigue, depressive disorder, anxiety, sleep disturbance, chronic illness, coronary heart disease, and reproductive disorders^4, 5^. Previous studies have reported that the LWH cause sustained hyperarousal (i.e., elevation of catecholamine levels or abnormal activation of the sympathetic nervous system) at work and even at rest^6, 7^. These conditions could exacerbate systemic inflammation and oxidative stress, which are risk factors for the abovementioned chronic illnesses^8^.

Periodontal conditions may portend systemic inflammation and oxidative stress^9^, as locally produced pro-inflammatory cytokines from the periodontal tissue can spread to target organs via systemic circulation^10^. Furthermore, periodontal disease is thought to be a risk factor for diabetes mellitus, cardiovascular disease, stroke, pulmonary disease, and adverse pregnancy outcomes^11–16^. The prevalence of periodontal disease worldwide ranges from 15% to 46%, and is 33% in South Korea^17–20^. The rate of severe periodontal disease is 8.9% in the United States^17^. Furthermore, according to the Global Burden of Disease 2015, prevalence of periodontal diseases was over 25% higher than 2005, disability-adjusted life year estimates of severe periodontitis was over 75% higher than 1990^21, 22^. Considering the high prevalence of periodontitis and its detrimental effect on the general population^23^, it is considered a public health problem^24^. Especially, working population could be vulnerable for periodontal disorders considering age distribution of severe periodontitis and lack of time to medical examination. The prevalence of most of adults’ oral disorders peaked after 70 years old, while that of severe periodontal disease peaked nearly 2 decades earlier^21^. A previous study indicated that workers with LWH have increased risks of forgoing healthcare needs because of the lack of time^25^. Hence, periodontitis may be aggravated by the lack of regular dental examinations^26^.

Therefore, investigating an association between periodontal disease and an undesirable work environment would be a major area of interest within the field of both occupational and dental health. Furthermore, linking LWH and strenuous shift work to periodontitis would reveal another factor contributing to the deterioration of health. While a recent study showed that undesirable work environments may adversely impact periodontal health^27^, too little is known about the relationship between undesirable work environments and periodontitis, particularly in terms of LWH.

The purpose of this investigation was to explore the relationship between LWH and periodontitis.

## Results

The baseline characteristics of participants with and without periodontitis are shown in Table 1. Of the 17,533 participants in this research, 5,201 had periodontitis (29.7%); the prevalence of periodontitis was significantly higher in males (36.1%) than in females (22.1%). A significant association was also found between the prevalence of periodontitis and age; with the oldest age group (51–65 years) having the highest prevalence (45.1%) and the youngest (19–30 years) having the lowest (5.3%). The prevalence of periodontitis was higher among individuals with lower educational and household income levels. Current smokers had a significantly higher prevalence of periodontitis (P < 0.0001). Those who drank alcohol heavily (34.5%) and those with abnormal sleep durations (31.1%) also showed higher rates of periodontitis, although with no statistical significance. The prevalence of periodontitis was lower among those who engaged in vigorous physical activity 3 days and more (27.3%) compared to those who engaged in less than 3 days or no activity per week. Central obesity, diabetes, hypertension, elevated triglycerides, and decreased high density lipoprotein cholesterol were associated with significantly higher rates of periodontitis. Daily oral care or preventive annual dental examinations were associated with significantly lower periodontitis rates. Green-collar, shift, and LWH workers showed significantly higher rates of periodontitis than other worker categories.

**Table 1:** Basic characteristics of study participants according to periodontitis status (n = 17,533).

	Periodontitis				
	Yes,		No		P Value
	n	%	n	%	
All	5,201	29.7	12,332	70.3	
Sex					<0.0001
	Male	3,425	36.1	6,058	63.9
	Female	1,776	22.1	6,274	77.9
Age (years)					<0.0001
	19–30	141	5.3	2,527	94.7
	31–40	728	18.5	3,214	81.5
	41–50	1,840	34.1	3,554	65.9
	51–65	2,492	45.1	3,037	54.9
Education					<0.0001
	Middle school	1,848	44.5	2,305	55.5
	High school	1,901	29.9	4,458	70.1
	More than college	1,452	20.7	5,569	79.3
House hold income					<0.0001
	1st Quartile	513	35.0	951	65.0
	2nd Quartile	1,421	33.1	2,879	66.9
	3rd Quartile	1,643	29.4	3,952	70.6
	4th Quartile	1,624	26.3	4,550	73.7
Smoking					<0.0001
	Never	2,057	22.9	6,931	77.1
	Former	641	31.0	1,427	69.0
	Current	2,503	38.7	3,974	61.4
Alcohol drinking					0.1829
	Never	1,039	32.4	2,172	67.6
	Moderate	3,202	27.8	8,334	72.2
	Severe	960	34.5	1,826	65.5
Vigorous physical activity (per a week)					0.0546
	None	3,072	30.0	7,181	60.0
	Less than 3 days	1,549	30.1	3,604	69.9
	3 days or more	580	27.3	1,547	72.7
Abnormal sleep duration (hours/day)					0.0634
	Yes (<6 or >8)	944	31.1	2,095	68.9
	No (6 ~ 8)	4,257	29.4	10,237	70.6
Chronic disorders					
Central obesity	1,895	35.4	3,456	64.6	<0.0001
Diabetes	1,929	41.4	2,731	58.6	<0.0001
Hypertension	806	44.1	1,023	55.9	<0.0001
Elevated triglycerides	1,933	37.6	3,202	62.4	<0.0001
Decreased high density lipoprotein cholesterol	1,763	33.3	3,531	66.7	<0.0001
Metabolic syndrome					<0.0001
	Yes	1,316	42.4	1,791	57.6
	No	3,885	26.9	10,541	73.1
Daily dental care					<0.0001
	Yes	2,604	26.4	7,278	73.7
	No	2,597	33.9	5,054	66.1
Annual dental examination					0.0084
	Yes	1,451	28.3	3,685	71.7
	No	3,750	30.3	8,647	69.7
Occupational classification					<0.0001
	White collar	1,397	20.6	5,373	79.4
	Pink-collar	1,102	27.7	2,873	72.3
	Green-collar	716	46.5	825	53.5
	Blue-collar	1,986	37.9	3,261	62.1
Employment status					<0.0001
	Paid workers	2,885	25.8	8,290	74.2
	Self-employed	1,924	36.8	3,306	63.2
	Another	392	34.8	736	65.2
Working schedule					<0.0001
	Fixed	2,665	26.8	7,292	73.2
	Shift	2,536	33.5	5,040	66.5
Working for over 40 hours per a week					<0.0001
	Yes	3,140	31.9	6,698	68.1
	No	2,061	26.8	5,634	73.2
Weekly working hours					<0.0001
	≤40	2,061	26.8	5,634	73.2
	40< and ≤52	1,350	28.9	3,324	71.1
	>52	1,790	34.7	3,374	65.3

The specific results of CPI according to oral sextant anatomy are presented in Supplementary Table 1.

The prevalence ratio (PR) and 95% confidence interval (CI) for periodontitis by working hours and CPI score are presented in Table 2. The prevalence of periodontitis was significantly associated with LWH (PR: 1.19, 95% CI: 1.14–1.24). Moreover, there were significant relationship between working hours and periodontitis, depending on increased working hours. The highest PR between periodontitis and LWH was founded in workers who working over 52 hours per week with CPI 4 group (PR: 1,46, 95% CI: 1.30–1.65).

**Table 2:** Prevalence ratio for periodontitis.

	Prevalence ratio (95% confidence interval)		
	Periodontitis	By Community Periodontal Index (CPI)	
		CPI 3	CPI 4
Long working hours	1.19 (1.14–1.24)	1.20 (1.13–1.27)	1.28 (1.15–1.42)
By Weekly work hours (hours)			
40< and ≤52	1.08 (1.02–1.14)	1.09 (1.02–1.17)	1.08 (0.95–1.23)
>52	1.29 (1.23–1.37)	1.31 (1.23–1.39)	1.46 (1.30–1.65)

The relationship between LWH and periodontitis according to our multiple logistic regression models are shown in Table 3. Among all participants, the LWH group was more likely to experience periodontitis after adjusting for all confounding factors. Both men and women in the LWH group were more likely to have periodontitis when stratified individually.

**Table 3:** Odds Ratio (OR) and 95% confidence interval (CI) for periodontitis according to long working hours with subgroup analysis.

	Long working hours (reference ≤40 hours/week)	
	OR	95% CI
Total participants (n = 17,533)	1.09	(1.02–1.18)
Gender		
Male workers (n = 9,483)	1.05	(0.95–1.15)
Female workers (n = 8,050)	1.16	(1.03–1.30)
Abnormal sleep duration		
Yes (n = 3,039)	1.17	(0.98–1.39)
No (n = 14,494)	1.08	(1.00–1.17)
Annual dental examination status.		
Yes (n = 5,136)	0.96	(0.84–1.10)
No (n = 12,397)	1.16	(1.06–1.26)
Daily oral care		
Yes (n = 9,882)	1.17	(1.06–1.29)
No (n = 7,651)	1.02	(0.92–1.13)
Vigorous physical activity (per a week)		
None (n = 10,253)	1.12	(1.02–1.23)
Less than 3 days (n = 5,153)	1.06	(0.93–1.21)
3 days or more (n = 2,127)	1.09	(0.88–1.36)
Smoking status		
Never (n = 8,988)	1.13	(1.01–1.26)
Former (n = 2,068)	1.02	(0.83–1.26)
Current (n = 6,477)	1.07	(0.95–1.20)
Alcohol drinking		
Never (n = 3,211)	1.17	(0.99–1.38)
Moderate (n = 11,536)	1.06	(0.96–1.16)
Severe (n = 2,786)	1.16	(0.96–1.39)
Working schedules		
Fixed-workers (n = 9,957)	1.19	(1.08–1.32)
Shift-workers (n = 7,576)	0.99	(0.89–1.10)

All models were adjusted for age, sex, socioeconomic status (educational level and household income level), occupational classification, job position, health behaviour (alcohol drinking, smoking, physical activity level, and daily sleep duration), chronic disorders (metabolic syndrome), and dental care (daily dental care and annually dental examination) except a stratified variable, respectively.

The correlations between periodontitis and the numbers of weekly work hours are presented in Table 4. In the total population, both the severe (over 52 hours per week) and moderate (41 to 52 hours per week) LWH groups showed higher periodontitis rates. There was a significantly increased risk of periodontitis according to LWH in those who did not undergo preventive annual dental health examinations (P for trend, 0.0028). However, there was no significant association between working hours and periodontitis among participants who did not care their oral health daily. The association between periodontitis and LWH after stratification according to smoking and drinking status were shown in Table 4 too. There were increased, but statistically non-significant association between periodontitis and working hours. Finally, when stratified by working schedule, a significant exposure-response relationship between periodontitis and working hours was only observed among subjects with fixed working schedules.

**Table 4:** Odds Ratio (OR) and 95% confidence interval (CI) for periodontitis by long working hours category with subgroup analysis.

	Weekly work hours (reference ≤40)				
	40< and ≤52		>52		P for trend
	OR	95% CI	OR	95% CI	
Total participants (n = 17,533)	1.09	(0.99–1.19)	1.10	(1.01–1.20)	0.0233
Gender					
Male workers (n = 9,483)	1.02	(0.91–1.14)	1.07	(0.96–1.19)	0.2276
Female workers (n = 8,050)	1.18	(1.03–1.37)	1.13	(0.98–1.30)	0.0439
Abnormal sleep duration					
Yes (n = 3,039)	1.11	(0.89–1.39)	1.21	(0.99–1.47)	0.0577
No (n = 14,494)	1.15	(1.04–1.28)	1.16	(1.05–1.28)	0.1007
Annual dental examination status.					
Yes (n = 5,136)	0.96	(0.81–1.13)	0.96	(0.81–1.24)	0.6066
No (n = 12,397)	1.18	(1.05–1.31)	1.23	(1.10–1.37)	0.0028
Daily oral care					
Yes (n = 9,882)	1.15	(1.02–1.30)	1.19	(1.05–1.34)	0.0040
No (n = 7,651)	1.01	(0.89–1.15)	1.03	(0.91–1.16)	0.6724
Vigorous physical activity (per a week)					
None (n = 10,253)	1.13	(1.00–1.27)	1.11	(0.99–1.24)	0.0521
Less than 3 days (n = 5,153)	1.05	(0.89–1.23)	1.07	(0.91–1.25)	0.4094
3 days or more (n = 2,127)	1.01	(0.78–1.32)	1.18	(0.91–1.53)	0.2504
Smoking status					
Never (n = 8,988)	1.17	(1.02–1.33)	1.09	(0.96–1.24)	0.1173
Former (n = 2,068)	1.00	(0.78–1.29)	1.04	(0.82–1.34)	0.7384
Current (n = 6,477)	1.02	(0.89–1.17)	1.11	(0.98–1.27)	0.1118
Alcohol drinking					
Never (n = 3,211)	1.29	(1.05–1.58)	1.08	(0.89–1.31)	0.2977
Moderate (n = 11,536)	1.04	(0.93–1.16)	1.07	(0.96–1.20)	0.2058
Severe (n = 2,786)	1.08	(0.87–1.34)	1.22	(0.99–1.50)	0.0566
Working schedules					
Fixed-workers (n = 9,957)	1.19	(1.05–1.34)	1.20	(1.06–1.34)	0.0017
Shift-workers (n = 7,576)	0.97	(0.85–1.11)	1.00	(0.88–1.13)	0.9986

All models were adjusted for age, sex, socioeconomic status (educational level and household income level), occupational classification, job position, health behaviour (alcohol drinking, smoking, physical activity level, and daily sleep duration), chronic disorders (metabolic syndrome), and dental care (daily dental care and annually dental examination) except a stratified variable, respectively.

## Discussion

We found that LWH in workers are associated with a significantly increased prevalence of periodontitis. Furthermore, this effect exhibited an exposure-response relationship even after subjects were adjusting for, and stratified according to, a number of covariates.

This is the first study to link the effect of excessive working hours, to periodontitis. However, our results are consistent with those of a previous study that found a relationship between shift work and periodontitis^27^. Nowadays, many individuals are exposed to the shift work and/or LWH; such substandard working conditions can interfere with workers’ recreation and rest periods, which are fundamental to one’s wellbeing^5^. Our results support the notion that engaging in LWH or abnormal schedules is related with decreased heath condition, with periodontitis being one of the signs.

There are several possible explanations for our results. Extended work can activate the hypothalamic-pituitary-adrenal (HPA) stress-response system, which is a possible precursor for periodontal disease as activation of the HPA axis stimulates the adrenal cortex to raise the level of glucocorticoids^28^. Work stress also stimulates the sympathetic nervous system to secrete catecholamines (norepinephrine and epinephrine) in the adrenal medulla. Stress-related hormones have been widely implicated in periodontal disease; previous studies revealed that patients with periodontal disease have above-normal concentrations of total urinary metanephrine (a metabolite of epinephrine) and salivary cortisol^29–31^. The stress-response system may also attenuate immunity, leading to periodontal tissue breakdown^32^.

Due to lack of time to recovery stress from work, the immune system might bring dysfunction by being not attentive enough so that increased chance to infectious agents (viruses and bacteria) enter the whole body and cause infectious disease^33^. Periodontitis is an inflammatory chronic disease of gingival tissue caused by bacterial infection^34^. Thus, periodontitis among works who worked long hours could be aggravated by increased vulnerability from infectious agents due to not enough time to recovery of work stress. Further researches are required to establish the association between LWH and periodontitis as an infectious disease.

Work-related stress can also associated with periodontal disease, as it can induce habits such as smoking, excessive alcohol consumption, and neglect of oral hygiene. Tobacco use is a widely-accepted risk and aggravating factor for periodontal disease^18, 32, 35^. It is well known that cigarette smoke exposure is a strong risk factor for periodontitis, as it reduces elastase and neutrophil levels while elevating T-cells in the oral cavity^36, 37^. In the literature on effects of alcohol consumption, the linkage of periodontitis is debated^38, 39^. Current research also indicated closed but non-statistical significant relationship between LWH and periodontitis according to drinking or smoking status. Further studies are needed to determine the association between alcohol consumption or smoking and periodontitis focused on working population.

We demonstrated that a lack of regular dental examinations is significantly linked to the association between periodontitis and LWH. A previous study examined the relationship between LWH and accessibility to hospital facilities^25^. Many workers with LWH could not meet their healthcare needs because of the lack of time. As periodontitis is one important early indicator of poor health^9^, it may be considered an identifier of workers-at-risk. Thus, there is a need for enhancing accessibility to regular medical or dental check-ups in the workplace.

Inadequate sleep is a potential risk factor for increased inflammatory and pro-inflammatory factors that could aggravate periodontal diseases^40^. However, previous research failed to show a significant correlation between short or long sleep durations and periodontitis^41^.

In current study, subjects who had vigorous physical activity at least 3days and more per a week showed decreased risk for periodontitis. Previous cross-sectional study using 12,110 general population also indicated that “5 and more episodes of moderate or 3 and more episodes of vigorous-intensity physical activity per week” related to lower periodontitis^42^. Furthermore, another cross-sectional study from Jordan with 340 participants indicated that low (38%) and moderated physical activity (28,7%) showed higher prevalence of periodontitis than high physical activity (13.1) which was measured by International Physical Activity Questionnaire (IPAQ, http://www. Ipaq.ki.se/ipaq.htm)^43^. Moderate physical activity from Jordan study same as our study’s 3 times and more vigorous physical activity level. In summary, the periodontitis might be protectable by at least 3 days and more vigorous physical activity. Nevertheless, without comprehensive assessment for the physical activity such as the IPAQ.

A previous study demonstrated a significant association between shift work and periodontitis^27^; our study showed a significant relationship between LWH and periodontitis only among fixed-scheduled workers, but not shift workers. Furthermore, there were no synergic or additive effects between work schedules and working hours on periodontitis (data not shown). To understand workers’ health statuses as related to the workplace, it may be helpful to use alternative analysis methods including stratification by undesirable working conditions such as LWH and shift work.

While this is the first study to investigate the relationship between LWH and periodontal diseases in the working population with a statistically strong power (17,517 participants), there are some limitations to our study. Our research was based in South Korea, which has the second-longest average working hours in OECD countries^44^; therefore, our results may not be generalizable. However, our findings are still highly relevant to understanding workers’ health because working more than 40 hours per week is clearly defined as LWH in many countries. Unfortunately, data from the KNHANES did not include advanced clinical approaches such as imaging and bone loss evaluation for diagnosing periodontitis. Periodontitis positivity in our study was only determined by using the CPI. Furthermore, dental examination for periodontitis was based on 10 index teeth. Previous investigators warned about the possibility of underdiagnosing periodontitis by not examining the mouth fully^45, 46^. Thus, results from current investigation of periodontal status linked to working hours were limited only positive of CPI as a screening test for periodontitis which was insufficient to reflect individuals’ periodontal status perfectly. Further investigations are required to estimate the association between working conditions and periodontitis using full-mouth examination.

Also, KNHANES had two information (daily oral care and annually dental examination) for oral health behaviors. Periodontal health status might be closely related with tooth brushing, flossing, other supplementary devices or specific oral conditions. Due to nature of data, we could demonstrate oral health behaviors status insufficiently.

Our findings may be somewhat limited by the cross-sectional nature of the KNHANES. study design. It difficult to determine a cause-and-effect relationship between LWH and periodontitis; thus, longitudinal investigations are required for this purpose.

In conclusion, our large, nationally representative study demonstrated a significant association between periodontitis and workers engaged in LWH. The current findings extend our knowledge of the adverse impacts of LWH to include maladies such as periodontitis. Future detailed investigations with longitudinal studies on the link between LWH and periodontal diseases will provide better insight into the effects of undesirable working condition on workers’ health.

## Methods

### Study participants

**Figure 1:**
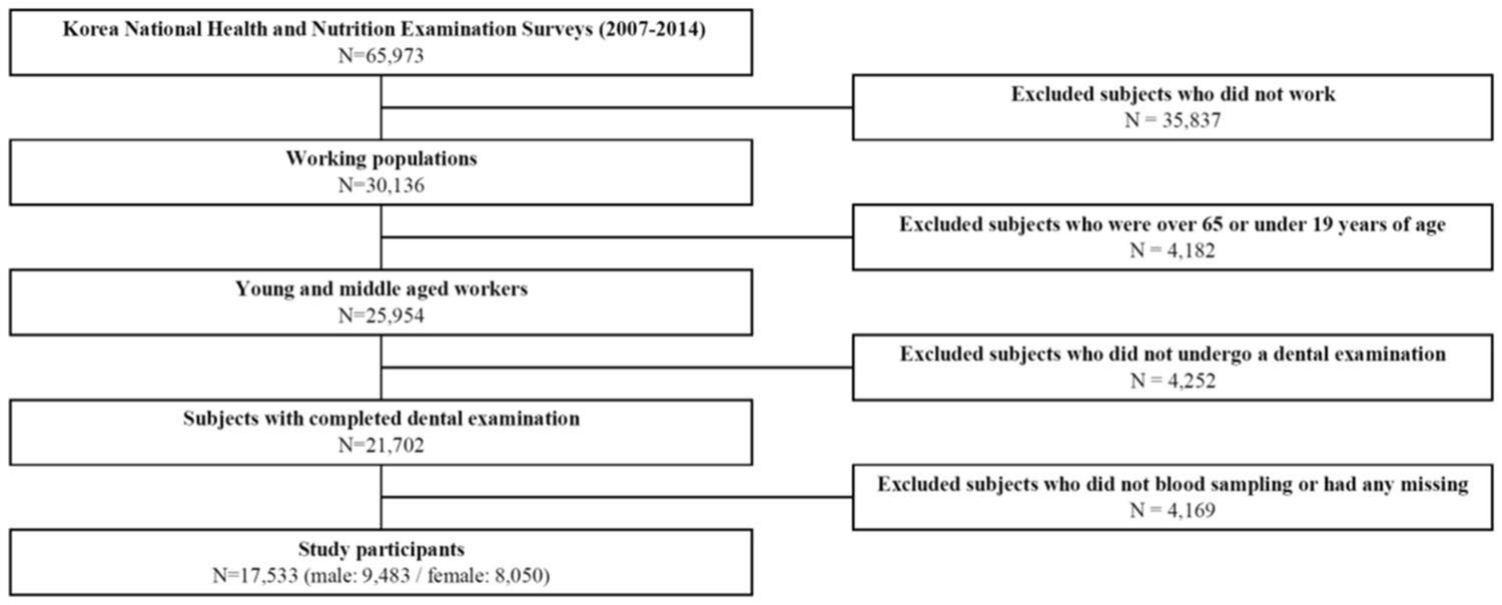
Schematic diagram depicting selection of study participants.

We utilized the KNHANES IV (2007–2009), V (2010–2012), and VI (2013–2014) survey data. The KNHANES are a series of nationally representative, cross-sectional, and population-based surveys on the health and nutritional status of Korean citizens; they are conducted annually by the Korea Centers for Disease Control and Prevention (KCDCP)^47^. For each survey, participants are newly chosen using proportional systematic sampling with multistage stratification based on residence, age, and sex by household registries in South Korea. Trained interviewers administered questionnaires on socioeconomic characteristics, health-related behaviour, and medical history either at the subjects’ home or at a mobile examination centre. The KNHANES 2007–2014 surveyed a total of 65,973 subjects (4,594 in 2007, 9,744 in 2008, 10,533 in 2009, 8,958 in 2010, 8,518 in 2011, 8,058 in 2012, 8,018 in 2013, and 7,550 in 2014). The inclusion/exclusion criteria are shown in Fig. 1. The final dataset consisted of 17,533 participants. This research was approved by the Institutional Review Board of the KCDCP (IRB: 2007–02-CON-04-P 2008-04EXP-01-C, 2009-01CON-03-2C, 2010-02CON-21-C, 2011-02CON-06-C, 2012-01EXP-01-2C, 2013-07CON-03-4C, and 2013-12EXP-03-5C) according to the Ethical Principles for Medical Research Involving Human Subjects, as defined by the Helsinki Declaration. All KNHANSE subjects understood about anonymity to protect themselves and potential risks and benefits with written informed consents before they participated survey.

### LWH and occupational characteristics

To assess the total number of weekly working hours, we relied on the following question in the self-reported questionnaire: “How many hours do you work at your job per week on average, including overtime but excluding meal times?” LWH are defined as exceeding the hours of full-time work as designated by the law. In Korea, weekly working hours are limited to 40 as specified by the Standard Labour Act. This Act allowed up to 12 hours of overtime with the agreement of all parties^25, 48, 49^. For our purposes, LWH was defined as working for >40 hours per week. To investigate whether an exposure-response association existed between LWH and periodontitis, working hours were categorized as ≤40, 40< and ≤52, and >52 (severe LWH) per week.

Occupational classifications were categorized into 4 groups based on the 10 major categories of the International Standard Classifications of Occupations: white collar workers (managers, professionals, technicians, and associate professionals), pink collar workers (clerical support, service, and sales workers), green collar workers (skilled agricultural, forestry, and fishery workers), and blue collar workers (crafts and related trades, plant and machine operators and assemblers, and elementary occupations)^50^. Employment status were divided into three categories; paid workers, self-employed, and another. Another category was included non-paid familial workers. Working schedules were binomially classified according to self-reported questionnaire responses. Those who usually worked during the daytime (06:00–18:00), evening hours (14:00–24:00), or night-time (21:00–08:00) were classified as having fixed schedules, while those who worked according to any other schedule (24 hours shifts, split shifts, or irregular shifts) were classified as shift schedule workers.

### Periodontitis and dental care

Dentists examined participants’ oral health and periodontal conditions using the Community Periodontal Index (CPI; modified based on the Community Periodontal Index of Treatment Needs or CPITN) created by the World Health Organization^51^. A CPI probe has a 0.5-mm ball tip and a black band 3.5–5.5 mm long that measures the depth of periodontal pockets. Ten index teeth (#11, #16, #17, #26, #27, #31, #36, #37, #46, and #47) were assigned to a sextant (upper right: #17–14, upper anterior: #13–23, upper left: #24–27, lower right: #47–44, lower anterior: #43–33, and lower left: #34–37) and examined respectively. If index teeth were extracted or could not be evaluated, the remaining teeth were checked at their sextants and the highest CPI was scored. The CPI was categorized as code 0 (healthy status), 1 (bleeding on probing, no gingival pockets), 2 (calculus present, no gingival pockets, black band of probe fully visible), 3 (periodontal pockets 4–5 mm), and 4 (periodontal pockets ≥6 mm). In this study, a code of 3 or 4 (periodontal pockets ≥4 mm) for at least 1 sextant was defined as positive for periodontitis.

Oral health can be affected by individuals’ oral care habits, and the KNHANES separately questions subjects about daily dental care and annual dental examinations. Daily dental care included questions such as whether teeth were brushed the day before; participants were also asked whether they had a regular dental check-up (not related to disease) in the past year.

### Other covariates

We also investigated socioeconomic and lifestyle factors related to both LWH and periodontitis, as people with lower socioeconomic status (i.e., educational and income levels) or behaviours adverse to health (smoking, heavy alcohol drinking, and lower physical activity level) may have an increased risk of periodontitis^52, 53^. Furthermore, LWH are also linked to socioeconomic status and health behaviour^54, 55^. Additional covariates were age (19−30, 31−40, 41−50, and 51−65 years), education level (below middle school, high school, or college/university), household income quartile, smoking (never, former, or current), alcohol drinking (never, moderate, or heavy), weekly vigorous physical activity day (none, less than 3 days, and 3 days and more), abnormal daily sleep duration (<6 or 8< vs. 6 ~ 8 hours), and metabolic syndrome.

Metabolic syndrome was diagnosed according to previous study based on National Cholesterol Education Program Adult Treatment Panel III criteria and the Korean Society for the Study of Obesity^50^. The presence of more than three of following five abnormalities were defined as a metabolic syndrome: (i) central obesity (waist circumference >90 cm in males or >80 cm in females); (ii) hypertension (blood pressure ≥130/85 mm Hg or antihypertensive drug treatment); (iii) hyperglycemia (fasting glucose level of serum ≥ 100 mg/dl or use of diabetes medication); (iv) high triglyceride (TG) levels (TG ≥ 150 mg/dl or specific treatment for elevated TG); or (v) low high density lipoprotein cholesterol (HDL-C) levels (HDL-C < 40 mg/dl in males and <50 mg/dl in females or the use of treatment for low HDL-C).

Household income was compared with the standard income level of Korean citizens, calculated using standardized classification by sex, residence, and age groups. Alcohol consumption was defined differently according to sex; heavy drinking was defined as at least 7 and 5 glasses of alcohol consumed more than twice per week by men and women, respectively.

We used physical activity status focused on vigorous physical activity. Vigorous physical activity level was defined as 20 minutes or more per a day of exertion that produces increased respiration, it were divided into three categories according to vigorous physical activity days per a week following previous studies^42, 43^.

### Statistical analysis

We analyzed data using the SAS 9.4 software (SAS Institute Inc., Cary, NC). First, the demographics of the study population and the prevalence of periodontitis were calculated. Chi-square tests were used to compare differences in baseline characteristics according to periodontitis. The PR and 95% CI were calculated with working hours and CPI stratification. The OR and 95% CI of the relationship between LWH and periodontitis were estimated using a logistic regression model. The model was adjusted for adjusted for age, sex, socioeconomic status (educational level and household income level), occupational classification, job position, health behaviour (alcohol drinking, smoking, physical activity level, and daily sleep duration), chronic disorders (metabolic syndrome), and dental care (daily dental care and annually dental examination) except a stratified variable (sex, abnormal sleep duration, annual dental examination, daily oral care, vigorous physical activity, smoking status, alcohol drinking, and working schedule), respectively. Next, we used weekly work hours as a categorical variable (≤40, 41–52, or >52 hours) to assess the exposure-response association with periodontitis using fully adjusted logistic models with subgroup analysis according to sex, abnormal sleep duration, annual dental examination, daily oral care, vigorous physical activity, smoking status, alcohol drinking, and working schedule.

## Electronic supplementary material

Supplementary table 1
