# Supplementary material for: Relationship between long working hours and periodontitis among the Korean workers
Source: Sci Rep. 2017 Aug 11;7:7967. doi: 10.1038/s41598-017-08034-6 (PMC5554150; doi:10.1038/s41598-017-08034-6)
Supplement: Supplementary file 1 — Supplementary table 1 [file 41598_2017_8034_MOESM1_ESM.doc]

**Title:** Relationship between long working hours and periodontitis among the Korean workers

**Authors**

Wanhyung Lee, M.D.^1,2,3^, Sung-Shil Lim, M.D. ^4^, Byurira Kim D.D.S.^5^, Jong-Uk Won, M.D., Ph.D.^1,2,3,6^, Jaehoon Roh, M.D., Ph.D.^1,2,3,6^, and *Jin-Ha Yoon, M.D., Ph.D.^1,2,3,6^

**Affiliations**

^1^The Institute for Occupational Health, College of Medicine, Yonsei University, Seoul, Korea ^2^Graduate School of Public Health, College of Medicine, Yonsei University, Seoul, Korea ^3^Incheon Worker’s Health Center, Incheon, Korea

^4^Severance Hospital, Health System, Yonsei University, Seoul, Korea

^5^ Severance Dental Hospital, College of Dentistry, Yonsei University, Seoul, Korea

^6^Department of Preventive Medicine, College of Medicine, Yonsei University, Seoul, Korea

***Correspondence to this address:**

Jin-Ha Yoon M.D., Ph.D.

The Institute for Occupational Health, Department of Preventive Medicine,

College of Medicine, Yonsei University,

50, Yonsei-ro, Seodaemun-gu, 6, Korea, [03722]

Tel: +82-2-361-5375; Fax: +82-2-392-8622

E-mail: [flyinyou@gmail.com](mailto:flyinyou@gmail.com)

| Supplementary table 1. Results of Community Periodontal Index (CPI) for periodontitis by oral anatomy | | |
| --- | --- | --- |
|  | CPI 3 | CPI 4 |
|  |
| Oral sextant anatomy with teeth number |  |  |
| Upper Right: #17-14 | 2,603 | 539 |
| Upper Anterior: #13-23 | 894 | 206 |
| Upper Left: #24-27 | 2,441 | 550 |
| Lower Right: #47-44 | 2,401 | 368 |
| Lower Anterior: #43-33 | 595 | 125 |
| Lower Left: #34-37 | 1,980 | 354 |
| CPI positive / total participants | 5,201 / 17,533 | |
|  | | |
